# Attending is not enough: Responding to targets is needed for across-trial statistical learning

**DOI:** 10.3758/s13414-024-02952-0

**Published:** 2024-08-30

**Authors:** Ai-Su Li, Dirk van Moorselaar, Jan Theeuwes

**Affiliations:** 1 Department of Psychology, Soochow University, Suzhou, China; 2 Institute Brain and Behavior Amsterdam, Department of Experimental and Applied Psychology, Vrije Universiteit Amsterdam, Amsterdam, the Netherlands; 3 William James Center for Research, ISPA-Instituto Universitario, Lisbon, Portugal

**Keywords:** Across-trial statistical learning, Motor response, Attentional bias, Sequence learning

## Abstract

Recent evidence shows that observers are able to learn across-trial regularities as indicated by faster responses to targets whose location was predicted by the target’s location on the preceding trial. The present study investigated whether responding to both targets of the pair, as was the case in studies thus far, was needed for learning to occur. Participants searched for a shape singleton target and responded to the line inside. There were two across-trial predicting-predicted regularities regarding target locations: if the target appeared at one specific location on a given trial, it would appear at another specific location on the next trial. Unlike previous experiments, for one of these regularity pairs a response was only needed on either the first or the second target in the pair. Experiment 1 showed that across-trial learning only occurred when responding was required to both targets of a pair. If the response to one target of a pair had to be withheld, no learning occurred. Experiment 2 showed that the absence of learning cannot be attributed to carry-over inhibition resulting from not having to respond. After learning across-trial contingencies, learning remained in place even when the response to the first target of the pair had to be withheld. Our findings show that the execution of the (arbitrary) simple key-press response for both trials of the pair was needed for across-trial statistical learning to occur, whereas solely attending target locations did not result in any learning.

**Supplementary Information:**

The online version contains supplementary material available at 10.3758/s13414-024-02952-0.

## Introduction

Our world is highly structured, full of various temporal and spatial regularities. When exposed to regularities embedded in an environment, humans are able to pick up regularity patterns through a process called statistical learning (SL) and make use of them to guide and facilitate performance (Bogaerts et al., 2020; Frost et al., 2019). For example, when shopping in a supermarket that you visit each week, you probably will not stop at each area to search for the products on your list because, after a few shopping experiences, you have learned the spatial layout of this supermarket. Instead, you probably directly go to items that you need following a fixed order, for example, fruits, vegetables, meat, drinks, bread, and miscellaneous groceries (as they are sequentially close to each other), which obviously makes the process more efficient and thus saves time.

The behavioral benefits due to SL of spatial regularities are ubiquitous in the context of laboratory visual search (see Theeuwes et al., 2022, for a recent review). For instance, attention becomes biased towards the location that has a higher probability of containing a target, leading to more efficient search (e.g., Geng & Behrmann, 2005; Xu et al., 2022). In addition to distributional regularities across space, there is also research investigating the spatial associations bound across trials during visual search (e.g., imagine you are searching for items in a refrigerator, when you find the area for milk, you might predict that the area for meat will also be nearby), concerning whole (i.e., all items)-target association (Ono et al., 2005; Thomas et al., 2018), distractors-target association (Ono et al., 2005), target-target association (Boettcher et al., 2022; Li et al., 2022; Li & Theeuwes, 2020; Toh et al., 2021), and salient distractor-distractor association (Li, Bogaerts, et al., 2023a; Yu et al., 2023). Relative to no learning of distractor-distractor or distractors-target associations, target-target associations are easier to learn and are used by participants as demonstrated by faster and/or more accurate response on predictable trials than unpredictable trials (Boettcher et al., 2022; Li et al., 2022; Li & Theeuwes, 2020; Toh et al., 2021). Despite this, arguably due to its dynamic nature, across-trial SL regarding target locations is limited to certain conditions and is overall less reliable than learning static regularities (e.g., conditions in which the target is more likely to appear at one specific location). For example, it was found that across-trial target location learning only occurred in conditions in which the target was a pop-out singleton but not when search was slow and serial (e.g., T among Ls task) (Li et al., 2022; Toh et al., 2021), probably because there was too much noise for learning to occur (i.e., extraction of target-target spatial association is more difficult because all elements have to be searched serially).

As learning of across-trial target-target spatial associations is relatively fragile, one may wonder how such learning differs from learning static regularities. It is typically assumed that learning of static regularities (e.g., targets or distractors appear with a higher probability at specific locations in space) is accomplished via dynamic weight changes within assumed spatial priority maps (e.g., Fecteau & Munoz, 2006; Theeuwes et al., 2022; Zelinsky & Bisley, 2015). Yet such a static priority landscape cannot account for the learning of target-target associations, where a target at one location dynamically primes enhanced priority at another location in space (Ekman et al., 2023; Li et al., 2024). This raises the question whether the formation of such across-trial spatial associations can be accomplished solely on the basis of attentional processing, or alternatively also requires the involvement of a response, which may in turn strengthen the subsequent memory trace (Olivers & Roelfsema, 2020).

Previous studies have shown that during passive viewing, participants can unintentionally extract spatially co-occurring items of complex scenes (e.g., Fiser & Aslin, 2001; Zhao et al., 2011) or temporally structured stimuli (e.g., Fiser & Aslin, 2002; Turk-Browne et al., 2005). Yet, it is conceivable that across-trial spatial associations that extend in time (i.e., the combination of two) is more complex than co-occurring items. It is also necessary to point out that previous studies investigating target-target association learning in visual search (Boettcher et al., 2022; Li et al., 2022; Li & Theeuwes, 2020; Toh et al., 2021) never explicitly manipulated the extent to which a response to an item was needed. Specifically, participants were asked to localize the target either via a button press (Toh et al., 2021) or via a mouse click on the target (Boettcher et al., 2022). It is thus possible that participants learned the predicted motor response associated with a given display instead of learning the across-trial spatial associations concerning the target. In Li and Theeuwes (2020) and Li et al. (2022), participants had to discriminate and respond to an arbitrary target feature which was completely random and could not be predicted nor prepared in advance, preventing participants from learning a specific sequential motor response. Nevertheless, in these two studies, a response to the attended item was required on each and every trial, making it impossible to determine the role of responding in across-trial SL.

Although the role of motor response in statistical learning has thus largely been ignored, there is some evidence indicating that responding may strengthen memory traces (see Olivers & Roelfsema, 2020, for a recent review). Notably, recent studies have shown that the involvement of motor response or action could enhance incidental memory even for task-irrelevant stimuli (e.g., Shimane et al., 2022; Yebra et al., 2019). For instance, Yebra et al. (2019) instructed participants to passively view a stream of grayscale objects and execute or withhold a button-press (i.e., Go/NoGo task) depending on the frame color of the picture. An unexpected recognition test in which participants needed to make a three-alternative forced-choice judgment (remember, familiar, or new) was performed 1 h or 1 day later. Critically, results showed that the background pictures that co-occurred with a target (Go) were recollected better than the pictures that co-occurred with a non-target (NoGo). Yebra et al. (2019) called this benefit *action-induced memory enhancement (AIME)*. Note, however, this study was about task-irrelevant episodic memory, not related at all to implicit SL.

**Fig. 1:**
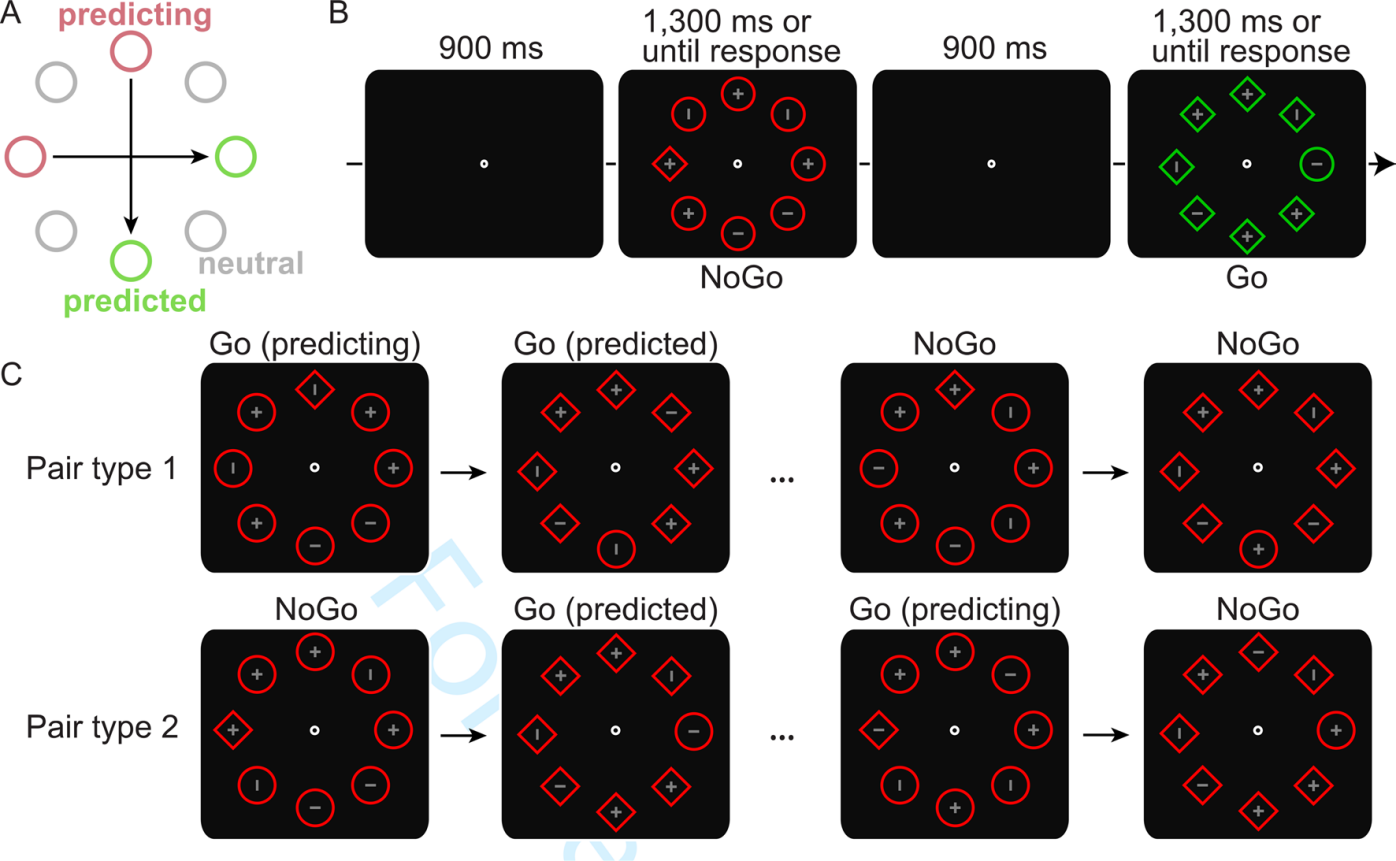
(**A**) Illustration of two regularity pairs of two spatial locations each. The target location on a preceding trial (labelled as predicting; denoted with red circles) predicted the target location on the subsequent trial (labelled as predicted; denoted with green circles). The neutral condition (denoted with gray circles) consisted of filler trials where the target appeared randomly at any other location. (**B**) Example of search displays where participants executed a discriminative button press if the shape singleton target contained a randomly assigned horizontal or vertical line (i.e., Go trials) but withheld their response if the shape singleton target contained a cross (i.e., NoGo trials). (**C**) Examples of two regularity pairs related to the response: one regularity pair had Go-Go and NoGo-NoGo combinations (each accounted for 50%) while the other regularity pair had NoGo-Go and Go-NoGo combinations (each accounted for 50%). Note both regularity pairs concerned the target location only, the target shape/color and line orientation inside (on Go trials) were completely random in real experiments

To determine the role of responding in SL, the present study combined the paradigm of Experiment 1 in Li and Theeuwes (2020) with a Go/NoGo task. Note that participants could only decide that it was a NoGo trial after attending the target. As illustrated in Fig. 1A, each participant was exposed to two across-trial regularities regarding target locations. The two regularity pairs were fixed and constant throughout the experiment, ensuring that participants were equally exposed to both pairs. Critically, we manipulated the predicting-predicted response type in different regularity pairs. As illustrated in Fig. 1C, for one regularity pair there were NoGo-Go and Go-NoGo combinations, while for the other regularity pair there were Go-Go and NoGo-NoGo combinations. This ensured that it was equally likely for predicting and predicted condition of both regularity pairs to be Go and NoGo (i.e., not any distributional response bias for any location). More importantly, such a manipulation made it possible to examine the role of responding in across-trial target location learning because during the processing of successive two target locations, the response was required for both targets of the pair if it concerned a Go-Go combination, while the response was only required for one target of the pair if it concerned a Go-NoGo or NoGo-Go combination. If motor responding plays a role in across-trial SL, the learning effect (i.e., faster response times (RTs) on predicted condition) should be dependent on response requirements. Alternatively, if responding is irrelevant, the learning effect should not differ.

## Experiment 1

### Method

#### Participants

The sample size was determined based on a simulation-based power analysis using simr R package (Green & MacLeod, 2016), following the similar procedure employed by previous work (e.g., Darda & Cross, 2022; Iniesta et al., 2021). We first collected data of 24 participants via Prolific (Palan & Schitter, 2018), which was exactly the same as the formal study. Subsequently, we fitted correct response times (RTs) without outliers of Go trials to a full model which was then simulated by extending the sample size to 40. Our focus was the interaction effect between prior trial type (NoGo vs. Go) and regularity (predicting vs. predicted) (for details see *Analysis*). The power simulations comparing the extended models with and without the fixed effect of interaction were run 200 times and suggested that a sample size of 40 participants was sufficient to detect the interested effect with a power of 83% (95% confidence interval (CI): 77.06, 87.93). Therefore, we continued to recruit participants via Prolific until we achieved the sample size of 40 (18 females and 22 males; *M*_age_ = 24.88 years, *SD*_age_ = 3.67). One participant was replaced because her overall accuracy of Go trials was below 75%. To preclude the possibility that the effect was influenced by the aware participants, we also replaced three participants who reported being aware of the sequential target locations across trials and correctly chose any predicted location (for details of awareness answers see Online Supplementary Materials (OSM)). All participants reported normal or corrected-to-normal visual acuity and gave informed consent before the experiment. The study was approved by the Ethics Committee of the Department of Experimental and Applied Psychology of Vrije Universiteit Amsterdam.

#### Apparatus

The experiment was created in OpenSesame (3.3.14) using OSWeb (Mathôt et al., 2012) and ran on a JATOS server (Lange et al., 2015). Participants were instructed to finish the task on their own computer or laptop in a quiet environment, with all possible distracting electronic devices off. The resolution specified in the experiment was 1,024 × 768 pixels (px). All stimuli were displayed on the “virtual monitor” in the center of the screen.

#### Procedure and design

The paradigm was modified based on Experiment 1 in Li and Theeuwes (2020). Figure 1B illustrates the trial sequence. At the beginning of each trial, a white (RGB: 255/255/255) fixation dot was presented at the center of the screen on a black background (RGB: 0/0/0) and remained visible throughout the trial. After 900 ms, the search array consisting of eight unfilled shapes (100 × 100 px, linewidth: 4 px) positioned on an imaginary circle (radius: 200 px) was presented for 1,300 ms or until the participant responded. Note, to have enough trials but also ensure the whole task was not too long (due to NoGo trials), we shortened the maximum RT of 2,000 ms as in Li and Theeuwes (2020) to 1,300 ms, which should still be a sufficiently long response window (i.e., average RTs of ~900 ms in the first block). All eight shapes were the same color, either green (RGB: 0/200/0) or red (255/0/0), with the same probability. The target shape was equally likely to be a diamond among seven circles or a circle among seven circles, and participants were instructed to search for the unique shape. On half of the trials (i.e., Go trials), the target shape contained either a horizontal or vertical gray (RGB: 128/128/128) line (16 px, linewidth: 2 px) with equal probability, and participants were asked to press the appointed response keys (“C” button for horizontal line, “M” button for vertical line) as fast and as accurately as possible. If participants did not respond correctly within 1,300 ms, they would receive a feedback message (i.e., “Your response was wrong!”) as well as an instruction screen repeating the correct key assignments for 800 ms. On the other half of trials (i.e., NoGo trials), the target shape contained a gray cross (16 × 16 px, linewidth: 2 px), which signaled that participants had to withhold their response. If a response was incidentally made, a text display “You should not respond to the cross” was shown for 800 ms. On any given trial, there were four shapes containing a cross and four shapes containing a line (two were horizontal and two were vertical). The target was evenly distributed at each location on both Go and NoGo trials, which were intermixed within each block. After each block, participants received the feedback consisting of accuracies of NoGo and Go trials as well as average RTs on Go trials in the preceding block. Breaks between blocks were controlled by participants themselves.

Like Li and Theeuwes (2020), each participant was exposed to two across-trial regularities regarding target locations. As illustrated in Fig. 1A, if on the prior trial the target was presented at the leftmost/top position of the display (predicting condition), it was always followed by the target presented at the rightmost/bottom position on the subsequent trial (predicted condition), or vice versa (counterbalanced across participants). Critically, we manipulated the predicting-predicted response type in different regularity pairs. As illustrated in Fig. 1C, for one regularity pair (horizontal or vertical, counterbalanced between participants) there were Go-Go and NoGo-NoGo combinations (each accounted for 50%), while for the other regularity pair there were NoGo-Go and Go-NoGo combinations (each accounted for 50%). This specific manipulation, which ensured that each position within a pair had the same probability of being a Go or a NoGo event (i.e., there were no distributional response biases), allowed us to investigate the modulation of response requirements on across-trial SL. Specifically, when forming an association between the successive target locations within a pair, a response was required to either both targets or none of the targets in one of the regularity pairs (Go-Go or NoGo-NoGo), while it was only required for one of the targets in the other regularity pair (NoGo-Go or Go-NoGo). If responding does not play a role, it is reasonable to assume that, for all individual combinations, participants should be able to learn the association between two targets of both pairs. In that case, the learning effect should not differ. However, if responding does play a role during learning, the pair with a Go-Go combination requires more responses (i.e., two) of the successive targets within a pair than the other pair (i.e., one). Then, the learning effect should be modulated by response requirements. Note this manipulation also ensured that the relative timing between the presentation of the targets in a pair was matched between the different regularity pairs (i.e., Go-Go matched Go-NoGo, NoGo-NoGo matched NoGo-Go). Both across-trial regularity pairs only concerned the target location while the target color, shape, and the line orientation inside (on Go trials) varied randomly across trials. The regularity pairs were randomly intersected among the neutral Go/NoGo trials in which the shape singleton target was presented randomly at one of the remaining four locations (Fig. 1A), with the constraint that the same across-trial location pair, regardless of response type, could not repeat back-to-back.

The experiment consisted of eight blocks of 128 trials each (full factorial design for target location, color, shape, line orientation, and response type), yielding 64 predicted Go/NoGo trials, 64 predicted Go/NoGo trials for each regularity pair, and 256 neutral Go/NoGo trials. Prior to the actual experiment, a practice block containing ten Go and ten NoGo trials in random order was repeated until the participant achieved the accuracy of 70% on both Go and NoGo trials. Participants were instructed to attain a minimum overall accuracy of 80% on both Go and NoGo trials during the formal blocks. After finishing the task, participants were asked whether they were aware of any sequential regularities regarding the target location such as one specific location was always followed by another. They also needed to answer two eight-alternative forced-choice questions (once for each regularity pair) indicating which location (displayed as a circular of eight white unfilled circles containing a number from 1 to 8) the target was most likely to appear after it was presented at the predicting location (illustrated by a white unfilled diamond among seven white unfilled circles) on the prior trial. All three questions were followed by a confidence judgment on a 5-point scale (1 = not certain at all, 5 = very certain).

#### Data analysis

The overall false alarm rate of NoGo trials was low (3.82%). The overall error rate (wrong responses) and miss rate (no responses) of Go trials were 6.07% and 4.84%, respectively. Correct (89.09%) RTs of each block of each participant were submitted to a non-recursive trimming procedure (Vanselst & Jolicoeur, 1994) that uses cell size to determine a criterion number of standard deviations (*SD*s) from the mean beyond which an observation is considered as an outlier (1.62%). Trials with RTs < 200 ms (0.01%) were also excluded from analysis.

Following Li et al. (2022), in addition to comparing predicting and predicted targets, we also analyzed neutral targets. By design, predicting and predicted targets were always separated by four items, and it is thus possible that any difference between these trials should not be attributed to learning of the spatial contingency, but instead simply results from a strategy, be it implicit or explicit, in which attention was discouraged to return to the previously inspected locations (inhibition of return; e.g., Klein, 2000; Li, Li et al., 2023b). Therefore, as in our previous work (Li et al., 2022), we ensured that we only included neutral trials, where the preceding target was also four positions away (i.e., matching the predicted condition). Note that such an across-trial target location distance could not be matched in the predicting condition, simply because the same regularity pair was never allowed to repeat.

As we were contrasting RTs (i.e., Go trials) as a function of whether the preceding trial required a response (Go) or not (NoGo), it is important to consider that responses are generally slowed when preceded by a NoGo event (e.g., Burnham, 2013; Koch & Philipp, 2005). An explorative analysis restricted to neutral and predicting trials confirmed that responses were slowed following NoGo trials, and this was the case irrespective of whether the specific response repeated or changed (see OSM). Given this post NoGo slowing it is crucial to take the prior trial type into consideration when contrasting predicting and predicted locations, and we therefore show predicting and predicted conditions as a function of whether the preceding trial required a response or not.

Using lme4 R package (Bates et al., 2015), we analyzed the RTs with linear mixed models (LMMs) (for error rate and miss rate analyses (see OSM)). We used mixed-effects models because they have multiple benefits over the traditional approach such as repeated-measures analysis of variance (RM-ANOVA). First, they treat the data at the trial level instead of the participant level so they retain richer information and can be particularly beneficial to noisy online experiments. Second, they can deal with the unbalanced design (Baayen et al., 2008) such as the present study in which the target appeared at different locations in different conditions with different trial numbers. Third, these approaches provide more power (Brysbaert & Stevens, 2018). All models were fitted with maximum likelihood and all included variables were dummy-coded.

For the first comparison, we included prior trial type (Go, NoGo), regularity (predicting, predicted), and their interaction as fixed effects. Target location (left, right, top, bottom) was included as an additional fixed effect to control for location specific biases (e.g., Huang et al., 2021; Xu et al., 2022). The random-effects structure was initialized with by-participan random intercepts as well as by-participant random slopes for prior trial type, regularity, and their interaction. To avoid overfitting of the random-effects structure, we used a principal component analysis (PCA; rePCA function in lme4) of random-effects variance-covariance estimates, and no component was removed. The *p*-values were obtained by the likelihood ratio test for comparing full models with and without the fixed effect of the Prior trial type × Regularity interaction. Planned pair-wise comparisons were performed using the emmeans R package. We reported estimate (*β*), standard errors (*SE*), degrees of freedom (*df*) for ratio statistics (*t*) as well as *p*-values. Next, restricting to trials with an across-trial target location distance of four items (an average of 17 neutral trials per prior trial type condition), we included prior trial type, regularity (predicted and neutral), and their interaction as fixed effects. The random-effects structure included by-participant random intercepts as well as by-participant random slopes for prior trial type and regularity.

### Results

**Fig. 2:**
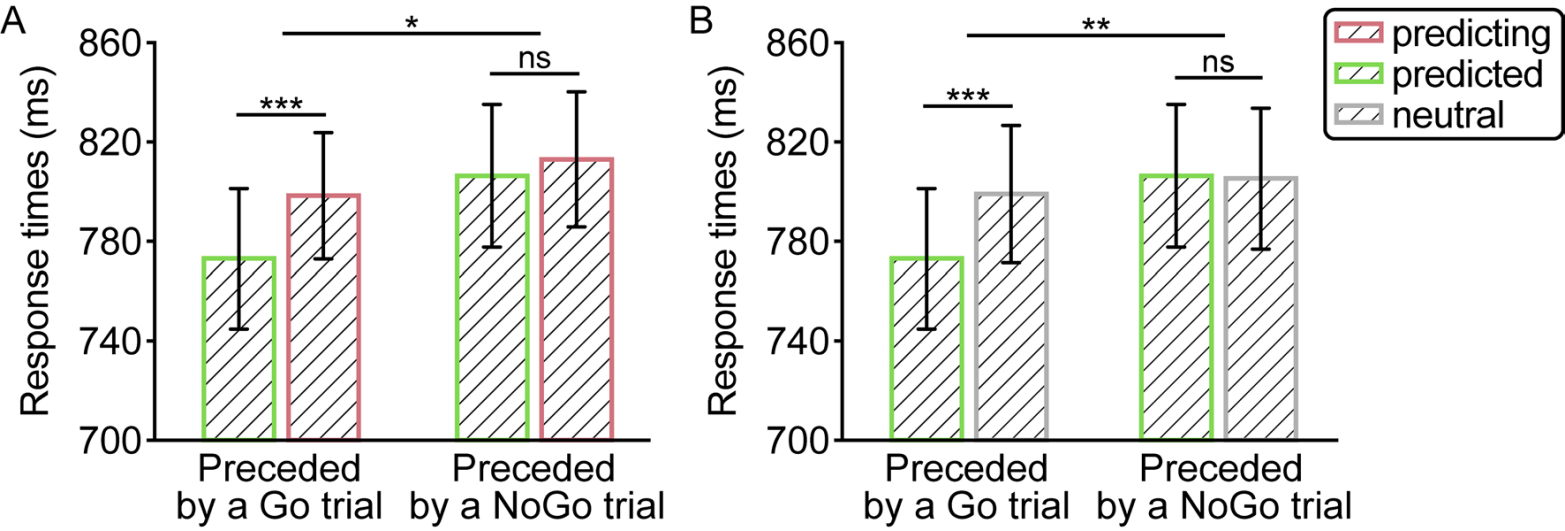
(**A**) Overall response times (RTs) as a function of prior trial type and regularity (predicted vs. predicting) in Experiment 1. (**B**) Overall RTs as a function of prior trial type and regularity (predicted vs. neutral) restricted to trials with an across-trial target location of four items in Experiment 1. The error bars denote 95% confidence intervals. *** *p* < .001, * *p* < .05, ns: not significant

As visualized in Fig. 2A, a reliable interaction [χ^2^(1) = 5.86, *p* = .015] reflected that, as in previous work by Li and Theeuwes (2020), predicted responses were faster than predicting responses [*β* = −25.64, *SE* = 5.61, *df* = 41.40, *t* = −4.57, *p* < .001], but critically only when the preceding trial also required a response. By contrast, when participants had to withhold their response on the preceding trial, the observed difference between predicting and predicted trials was no longer significant [*β* = −6.58, *SE* = 6.40, *df* = 41.20, *t* = −1.03, *p* = .31], suggesting that having to respond is indeed a critical factor in establishing across-trial SL. When limiting the analysis to those trials with an across-trial target location distance of four items (i.e., predicted vs. neutral), as visualized in Fig. 2B, a significant interaction effect was also observed [χ^2^(1) = 9.60, *p* = .002], reflecting that following Go trials, responses were faster at predicted than those at comparable neutral locations [*β* = −26.18, *SE* = 6.55, *df* = 123.00, *t* = −4.00, *p* < .001], replicating Li and Theeuwes (2020), but this difference disappeared following NoGo trials [*β* = 0.63, *SE* = 6.60, *df* = 129.00, *t* = 0.10, *p* = .92].

### Discussion

Experiment 1 showed that across-trial learning only occurred when responding was required to both targets of a pair. Critically, when the response to either target of the pair had to be withheld, participants did not respond faster to the second target of the pair, which suggests that no learning occurred. However, before we can conclude that responding is needed for across-trial association learning, we have to rule out an alternative explanation of why responding to the second target in a NoGo-Go pair may have been relatively slow. It is possible that there was learning; yet, responding to the second target may have been relatively slow because withholding a response to the first target caused some motor inhibition that carried over to the second target. In other words, the requirement of not responding to the first target may have masked a possible learning-induced speed-up of the response to the second target.

Experiment 2 was designed to test this alternative possibility. In Experiment 2 the same target location associations were used as in Experiment 1, but initially we introduced a training phase, in which both regularities had Go-Go and NoGo-NoGo combinations to ensure that they were able to learn these regularities (as in Experiment 1 and our previous work (Li et al., 2022; Li & Theeuwes, 2020)). After learning, the pair combinations switched to NoGo-Go and Go-NoGo ones in the test phase. The idea is that if withholding a response to the first target would mask a possible learning effect, then we should not observe response benefits to the second target during the test phase. If, however, the learning effect persists during the test phase, we can conclude that carryover motor inhibition from the preceding NoGo trial is not sufficient to offset the facilitation effect to the second predicted target and hence the regularity pair with NoGo-Go and Go-NoGo combinations in Experiment 1 was indeed not learned.

## Experiment 2

Experiment 2 was identical to Experiment 1 except that the response combinations of regularity pairs were manipulated in different blocks. During the first three blocks (training phase), both regularity pairs had Go-Go and NoGo-NoGo combinations, ensuring robust learning (Li et al., 2022; Li & Theeuwes, 2020). During the subsequent five blocks (test phase), response combinations of regularity pairs switched to NoGo-Go and Go-NoGo ones, allowing us to examine whether learning, once in place, would persist when there might be carry-over motor inhibition from withholding the response to the preceding NoGo trial.

### Method

The method was identical to that of Experiment 1, with the following changes: First, a new set of 40 participants (15 females and 25 males, *M*_age_ = 23.38 years, *SD*_age_ = 2.67) were recruited via Prolific. Two participants whose accuracy of Go trials was below 75% and another three participants who reported being aware of sequential target location regularities and correctly chose any predicted location were replaced. Second, both two regularity pairs were Go-Go and NoGo-NoGo combinations in the first three blocks (training phase) but switched to NoGo-Go and Go-NoGo combinations in the following five blocks (test phase).

#### Analysis

The overall false alarm rate of NoGo trials was low (4.44%). The overall error rate and miss rate of Go trials were 6.67% and 5.21%, respectively. RTs were limited to Go trials with correct responses (88.12%). The same non-recursive trimming procedure as in Experiment 1 was used for outlier removal (1.59%). There were no trials with RTs < 200 ms. Here we only reported RTs (see OSM for error rate and miss rate analyses). For the comparison between predicting and predicted conditions, LMM included target location, prior trial type, phase, regularity, and Phase × Regularity as fixed effects and included by-participant random intercepts, by-participant random slopes for phase, regularity and Phase × Regularity interaction as random-effects. The effect of interest was whether the learning persisted in the test phase, so pair-wise comparisons between predicting and predicted conditions in training and test phases were directly performed. Next, restricting to trials with an across-trial target location distance of four items (~six neutral trials per prior trial type condition in the training phase, and ~11 neutral trials per prior trial type condition in the test phase), we included prior trial type, phase, regularity, and Phase × Regularity interaction as fixed effects. The random-effects structure included by-participant random intercepts, by-participant random slopes for phase, regularity and Phase × Regularity interaction.

### Results

**Fig. 3:**
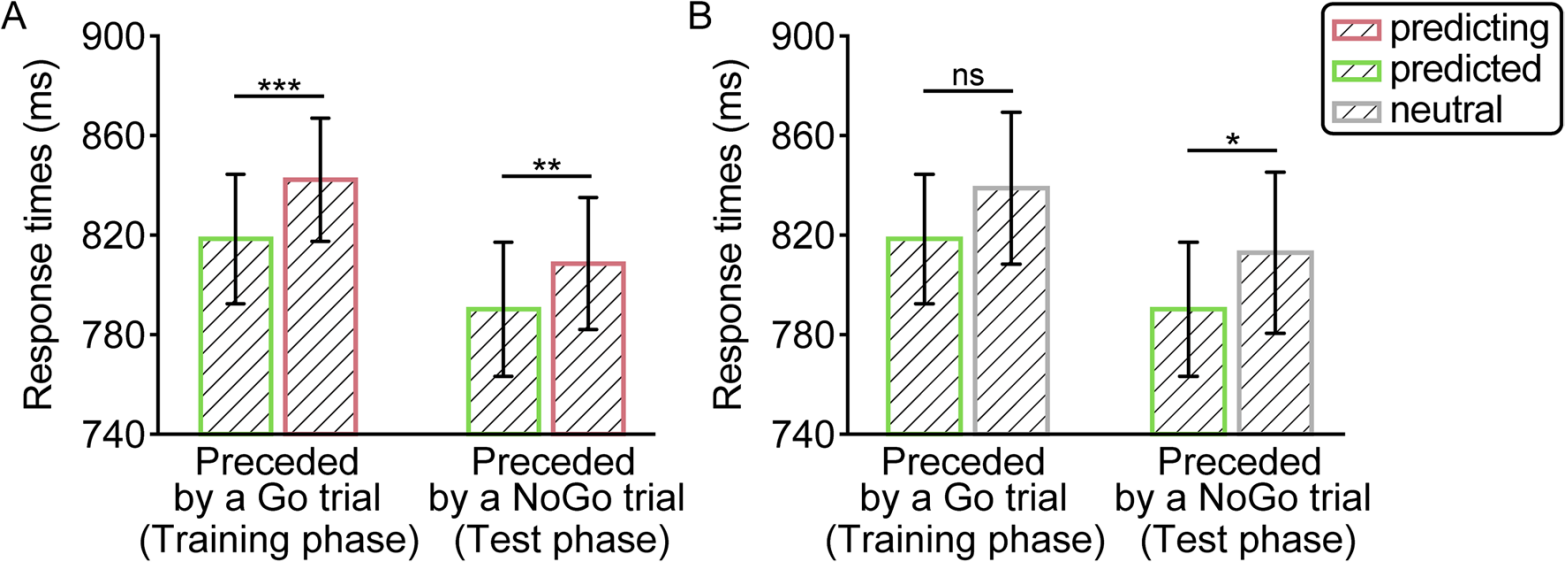
(**A**) Overall response times (RTs) as a function of regularity in the training and test phase of Experiment 2. (**B**) Overall RTs as a function of regularity restricting to trials with an across-trial target location of four items during the training and test phase of Experiment 2. The error bars denote 95% confidence intervals. *** *p* < .001, ** *p* < .01, * *p* < .05, ns: not significant

As a first step, we analyzed whether the training phase, where participants responded to both events in a pair, resulted in reliable learning. As shown in Fig. 3A, replicating Experiment 1, and Li and Theeuwes (2020), responses on predicted trials were reliably faster than on predicting trials [*β* = −24.00, *SE* = 6.09, *df* = 186.10, *t* = −3.94, *p* < .001]. Critically, this across-trial learning effect remained in place during the test phase of the experiment where on a subset of trials participants had to withhold their response during the preceding trial [*β* = −18.00, *SE* = 5.20, *df* = 97.80, *t* = −3.47, *p* = .001], an effect that did not change when the predicted trials were directly contrasted to matched neutral trials in terms of across-trial target location distance [*β* = −18.50, *SE* = 7.63, *df* = 69.60, *t* = −2.43, *p* = .018], as visualized in Fig. 3B. It should be noted that while numerically the same effect was also present when contrasting predicted and neutral trials following Go trials [*β* = −12.90, *SE* = 8.55, *df* = 59.90, *t* = −1.51, *p* = .14], it failed to reach significance, arguably to the low trial count in the neutral condition (~six trials).

### Discussion

This experiment shows across-trial learning, once established, remains in place when response has to be withheld during the preceding event, suggesting little evidence for response inhibition carryover effects. If anything, the results show that these response inhibition carryover effects (if they exist) are not strong enough to mask the learned across-trial facilitation effect. On the basis of this finding, we can conclude that the absence of learning of across-trial associations as found in Experiment 1 should be attributed to withholding the responses on either the predicting or the predicted target.

## General discussion

The present study investigated the role of responding in across-trial SL by manipulating the different response requirements of successive targets presented within a pair. Consistent with the idea that motor responses play a critical role in forming spatial associations across trials, our results show that target-target spatial association can only be learned when participants need to respond on both paired trials but not when the response on either the predicting or predicted trial needs to be withheld.

Although the results convincingly demonstrate that motor responses are a critical condition to extract across-trial target spatial associations, we can only speculate about the underlying mechanism. Much neural evidence suggests that medial temporal lobe (MTL), especially the hippocampus, plays a critical role in extracting sequential or spatially co-occurring events (e.g., Chun & Phelps, 1999; Fortin et al., 2002; Schapiro et al., 2012). One explanation is that the engagement of motor responses on both paired trials strengthens the memory trace of the two consecutive target locations (Yebra et al., 2019). Another explanation is that response inhibition and memory encoding compete for limited cognitive resources such that the suppression of a motor response may lead to a reduced memory encoding (Chiu & Egner, 2015). Regardless of memory enhancement on Go trials or memory diminishment on NoGo trials, the involvement of motor response consolidates the memory trace of target locations across trials and leads to the learning. Recent studies suggested a much stronger link between attention and action than previously assumed, indicating a tight coupling and bidirectionally link (see Olivers & Roelfsema, 2020, and van Ede, 2020, for recent reviews). The classic work by Allport (1987) proposed the framework of “selection for action,” suggesting that the function of selecting stimuli is to be able to take an action. Olivers and Roelfsema (2020) have put forward a new framework that integrates attention, memory, and action (see their Fig. 1). In this model, attention reflects the selective coupling of a task-relevant representation to a task-relevant action plan, and the link to action creates a feedback loop which in turn reinforces the selected representations, giving rise to faster selection, more robust memory, and more rapid retrieval in subsequent trials or tasks. The current findings are consistent with such a framework: only the target that led to an action outcome was reinforced such that responding sequentially to both targets resulted in the bounding and consolidation of the locations where these targets were presented.

The current findings can also be related to the BRAC (Binding and Retrieval in Action Control; Frings et al., 2020) framework in which it is assumed that the presentation of the target at a specific location is coded together with the response as a particular “event” (see also Theeuwes et al., 2024). It is conceivable that if no response needs be given, there is no coding of the location of the target as specific “event” and therefore there may be no learning.

The present study is relevant to the field of perceptual sequence learning. Across-trial SL in essence is sequence learning of stimulus-stimulus (S-S) association because the response to any given display is unpredictable. This differs from the sequence learning investigated with a traditional serial response time (SRT) task where participants responded to the target location (out of four locations) on the screen with a spatial compatible key (Nissen & Bullemer, 1987) involving S-S and response-response (R-R) as well as S-R association (see Schwarb & Schumacher, 2012, for a review). Compared to motor learning, S-S sequence learning (e.g., perceiving the stimulus without an immediate response) is more difficult to observe and occurs more often with participants who are aware than with participants who are unaware (e.g., Russeler & Rosler, 2000; Willingham, 1999). Remillard (2003) proposed a pure S-S sequence learning paradigm where participants respond to the identity (i.e., unrelated to response) of an underlined target among non-underlined distractors. This study shows that participants respond faster to the target location that had a higher probability of following the predicting target location than the paired target location with a lower probability. Remillard (2003) proposed that automatic orienting of attention was sufficient for pure perceptual sequence learning to occur. The current findings provide an important addition: despite its perceptual nature, S-S sequence learning also requires the involvement of a motor component.

As an alternative, one could argue that learning of the pair with the Go-Go combination may not be related to responding but instead may be due to a shorter temporal distance between the processing of two targets within a pair. Indeed, when a response is needed to both targets, the processing of the two targets may seem to be closer in time because the trial is terminated as soon as a response is given to the target of a pair. After an interstimulus interval (ISI) of 900 ms, the next trial is presented, which has the second target of the pair. In conditions where a response is only needed for the second target of a pair, the first target (with a NoGo response) remains present until time-out, i.e., after 1,300 ms the display is extinguished. Because of this time-out, more time passes between the presentation of targets in a NoGo-Go combination than in a Go-Go combination, and one could hypothesize that this time difference is responsible for the difference in learning. While this explanation may seem reasonable, it does not hold because the relative timing between two targets was matched between the two pairs (Go-Go matched NoGo-Go). If it was the shorter temporal distance that resulted in learning, the Go-NoGo combination should have been learned equally well and we should have been able to detect the response benefit on predicted Go trials of the NoGo-Go combination. Indeed, if learning did occur, the response benefit should generalize to both combinations. Therefore, the absence of a learning effect for the pair with NoGo-Go and Go-NoGo combinations has to be a consequence of no learning of either combination, providing strong evidence that responding to both items of the pair was needed for across-trial SL.

It is important to realize that the current study shows that spatially attending two sequentially presented target singletons is not enough for learning to occur. While no learning was observed, one still has to assume that the target singleton on the predicting or predicted trial had to be attended because the plus sign within the target singleton needed to be identified, indicating a NoGo trial. This implies that even though the target was attended in all conditions, across-trial learning occurred only when responding was required to both targets of a pair. One could speculate that attending alone is not enough for consolidating the location of the target into working memory, and therefore no learning could occur. When responding is required there is memory consolidation allowing learning of the across-trial association. This idea that attending information may not be enough for consolidating it into working memory is consistent with the recent notion of what has been labelled “memory re-selection” (Fu et al., 2023).

In sum, spatially attending to two sequentially presented target singletons is not sufficient for across-trial learning to occur. The execution of the (arbitrary) simple key-press response to each of two sequentially presented target singletons is needed to learn and establish an across-trial contingency.

## Supplementary Information

Below is the link to the electronic supplementary material.

Supplementary file1 (DOCX 347 KB)

## Data Availability

Data and materials for all experiments are available at: https://github.com/AisuLi/SL_across-trial_response, and none of the experiments was preregistered.

## Code availability

Code for all experiments is available at: https://github.com/AisuLi/SL_across-trial_response.
