# Supplementary material for: Attending is not enough: Responding to targets is needed for across-trial statistical learning
Source: Atten Percept Psychophys. 2024 Aug 30;86(6):1963–73. doi: 10.3758/s13414-024-02952-0 (PMC11410895; doi:10.3758/s13414-024-02952-0)
Supplement: Supplementary file 1 — Supplementary file1 (DOCX 347 KB) [file 13414_2024_2952_MOESM1_ESM.docx]

**Attending is not enough: responding to targets is needed for across-trial statistical learning**

(Supplementary Materials)

Consistent with analyses reported in the main text, we analyzed the RT data of Go trials with correct responses and without RT outliers.

**Experiment 1**

*Across-trial task repetition benefit/switch cost*

Previous studies have shown that RTs on Go trials that were preceded by a NoGo trial were slower than those preceded by a Go trial, suggesting an across-trial task repetition benefit or switch cost (e.g., Burnham, 2013; Koch & Philipp, 2005). To verify this effect, we analyzed unpredictable (predicting and neutral) trials without inter-trial target location priming (irrespective whether the prior trial was Go or NoGo). Prior response relationship (Go_same, Go_different, NoGo) was included as the fixed effect and by-participants random intercepts as well as by-participants random slopes for prior trial response relationship were included as the random effects. Results showed that trials with a different response were not reliably slower compared to the trials that had the same response as the previous trial, *β* = 4.33, *SE* = 5.22, *t* = 0.83, *p* = .41; yet, RTs on trials that were preceded by a NoGo trial were significantly slower, β = 17.10, *SE* = 5.64, *t* = 3.03, *p* = .004, suggesting that the benefit was due to the repetition of task instead of response.

*Awareness of the regularities*

To preclude the possibility that our effect of interest was influenced by participants that noticed the regularity, the three participants who reported to be aware of the sequential target locations across trials and correctly identified any of the predicted locations were replaced. Three out of 40 participants in the final dataset reported being aware of the sequential target locations with a mean confidence score (CS) of 3.67*,* but nevertheless failed to correctly identify the predicted locations (CS: 3.00). The remaining 37 participants reported to be unaware of the across-trial target location regularities (CS: 3.19), with a mean CS of 2.05.

*Error rate and miss rate of Go trials*

The maximum response time for Go trials was only 1.3 s, so the error rate (i.e., incorrect responses) and miss rate (i.e., no responses) were analyzed separately with generalized linear mixed models (GLMMs). We first conducted a comparison between predicting and predicted conditions. For both error and miss rate analyses, we included prior trial type (Go, NoGo), regularity (predicting, predicted), and their interaction as well as target location as fixed effects. The random-effects structure initialized with by-participants random intercepts as well as by-participants random slopes for prior trial type, regularity and their interaction. Using rePCA function in lme4, for the error rate analysis, we removed by-participants random slopes for regularity and Prior trial type × Regularity interaction. Full models with and without the fixed effect of Prior trial type × Regularity interaction were compared. In the error rate analysis, there was no significant Prior trial type × Regularity interaction, χ^2^(1) = 0.75, *p* = .38, as visualized in the left panel of Figure S1A. The pair-wise comparisons showed that the error rates on predicted condition were not different from predicting condition, neither when preceded by a Go [*β* = −0.18, *SE* = 0.13, *z* = −1.43, *p* = .15], nor by a NoGo trial [*β* = −0.03, *SE* = 0.11, *z* = −0.29, *p* = .77]. For the miss rate analysis, by-participants random slopes for prior trial type and Prior trial type × Regularity interaction were removed from the random-effect structure. As visualized in the right panel of Figure S1A, the model comparison also did not reveal a significant interaction, χ^2^(1) = 3.44, *p* = .064. In line with the RTs analysis, the pair-wise comparisons showed that when preceded by a Go trial, participants missed less trials in predicted condition than predicting condition [*β* = −0.42, *SE* = 0.17, *z* = −2.44, *p* = .015], and this difference disappeared if the preceding trial was a NoGo trial [*β* = −0.03, *SE* = 0.16, *z* = −0.20, *p* = .84].

Figure S1. (A) Error rate (wrong responses) and miss rate (no responses) as a function of prior trial type and regularity (predicted vs. predicting) in Experiment 1. (B) Error rate and miss rate as a function of prior trial type and regularity (predicted vs. neutral), restricting to trials with an across-trial target location distance of 4-item in Experiment 1.

The same analyses were repeated restricted to trials with an across-trial target location distance of 4 items, For both error rate and miss rate analyses, we included prior trial type (Go, NoGo), regularity (neutral, predicted), and their interaction as fixed effects. For the error rate analysis, we included by-participants random intercepts as well as by-participants random slopes for prior trial type and regularity as random effects. Full models with and without the fixed effect of Prior trial type × Regularity interaction were compared. We did not observe a reliable interaction, χ^2^(1) = 0.72, *p* = .39, as can be seen in the left panel of Figure 1B. The pair-wise comparisons showed that the error rate on predicted condition was not different from predicting condition, no matter whether they were preceded by a Go trial [*β* = −0.25, *SE* = 0.20, *z* = −1.25, *p* = .21], or preceded by a NoGo trial [*β* = −0.03, *SE* = 0.19, *z* = −0.17, *p* = .87]. For the miss rate analysis, we included by-participants random intercepts as well as by-participants random slopes for regularity as random effects. As visualized in the right panel of Figure S1B, the model comparison again did not reveal a significant interaction, χ^2^(1) = 1.93, *p* = .16. The pair-wise comparisons showed that when preceded by a Go trial, participants missed less trials in predicted conditions than predicting condition [*β* = −0.50, *SE* = 0.24, *z* = −2.08, *p* = .037], but this difference did not reach the significance when following a NoGo trial [*β* = −0.10, *SE* = 0.24, *z* = −0.42, *p* = .67]. Note that the total trial counts per prior trial type condition were 64 in the predicted condition and approximately 20 in the neutral condition.

**Experiment 2**

*Awareness of the regularities*

Three participants who reported to be aware of sequential target location regularities and correctly chose any predicted location were replaced. Out of the included 40 participants, there were two participants who reported being aware of the across-trial target association (CS: 3.5) but failed to identify any of the predicted locations (CS: 2.50). The remaining 38 participants reported to be unaware of the across-trial target location regularities (CS: 3.23), with a mean CS of 2.05 regarding locations they chose.

*Error rate and miss rate of Go trials*

Predicting vs. Predicted: For both error rate and miss rate analyses, GLMMs included target location, prior trial type, phase, regularity and Phase × Regularity as fixed effects. For the error rate analysis, we included by-participants random intercepts, by-participants random slopes for Phase as random effects while for the miss rate analysis, by-participants random intercepts as well as by-participants random slopes for phase, regularity and their interaction were included as random effects. As the question was that whether the learning effect persisted during the test phase, pair-wise comparisons between predicted and predicting conditions in two phases were directly conducted. As can been in the left panel of Figure S2A, there was no reliable difference between conditions in error rates during both training [*β* = −0.08, *SE* = 0.13, *z* = −0.57, *p* = .57] and test [*β* = −0.21, *SE* = 0.12, *z* = −1.76, *p* = .08] phases. Participants missed less trials in predicted condition than predicting condition during the training phase [*β* = −0.53, *SE* = 0.19, *z* = −2.85, *p* = .004], as shown in the right panel of Figure S2A, and there was no difference between the two conditions in the test phase [*β* = −0.25, *SE* = 0.22, *z* = −1.17, *p* = .24].

**

Figure S2. (A) Error rate and miss rate as a function of phase and regularity (predicted vs. predicting) while controlling for the prior trial type in Experiment 2. (B) Error rate and miss rate as a function of phase and regularity (predicted vs. neutral) while controlling for the prior trial type, restricting to trials with an across-trial target location distance of 4-item in Experiment 2.

Predicted vs. Neutral (trials with an across-trial target location distance of four items): For both error rate and miss rate analyses, GLMMs included prior trial type, phase, regularity and Phase × Regularity as fixed effects and included by-participants random intercepts, by-participants random slopes for Phase as random effects as random effects. As can seen in the left panel of Figure S2B, the error rate in predicted condition was not significantly different from neutral condition during both training [*β* = 0.11, *SE* = 0.21, *z* = 0.53, *p* = .59] and test [*β* = −0.21, *SE* = 0.18, *z* = −1.13, *p* = .26] phases. As shown in the right panel of Figure S2B, the miss rate for predicted targets was not significantly different from neutral targets during both training [*β* = −0.26, *SE* = 0.23, *z* = −1.14, *p* = .25], and test [*β* = 0.19, *SE* = 0.24, *z* = 0.78, *p* = .43] phases. Note that the total trial counts per prior trial type condition of predicted and neutral trials were 48 and ~8 in the training phase, while they were 80 and ~13 in the test phase.

**References**

Burnham, B. R. (2013). Response retrieval in a go/no-go priming-of-popout task. *Psychonomic Bulletin and Review*, *20*(6), 1187-1194. <https://doi.org/10.3758/s13423-013-0433-0>

Koch, I., & Philipp, A. M. (2005). Effects of response selection on the task repetition benefit in task switching. *Memory and Cognition*, *33*(4), 624-634. <https://doi.org/10.3758/bf03195329>
